# Natural History of Untreated Retinoblastoma

**DOI:** 10.3390/cancers13153646

**Published:** 2021-07-21

**Authors:** Junyang Zhao, Zhaoxun Feng, Brenda L. Gallie

**Affiliations:** 1 Department of Ophthalmology, Chongqing Aier Children’s Eye Hospital, Chongqing 400020, China; zhaojunyang@163.com; 2 Department of Ophthalmology, University of Ottawa, Ottawa, ON K1H 8M2, Canada; zfeng100@uottawa.ca; 3 Department of Ophthalmology, Hospital for Sick Children, Toronto, ON M5G 1X8, Canada; 4 Krembil Research Institute, Toronto, ON M5T 0S8, Canada; 5 Techna Institute, Toronto, ON M5G 1L5, Canada; 6 Department of Ophthalmology, Medical Biophysics, Molecular Genetics, University of Toronto, Toronto, ON M5T 3A9, Canada

**Keywords:** retinoblastoma, natural history, death, mortality, prognosis, treatment abandonment

## Abstract

**Simple Summary:**

Treatment abandonment is a leading cause of mortality for children with retinoblastoma worldwide. Noncompliance is influenced by both clinical and social factors including parents’ lack of appreciation of the aggressive nature of the disease. The natural history of retinoblastoma has not been previously studied because it is unethical to subject children with active cancer to nonintervention, and children who abandoned treatment were often lost to follow-up. Through phone interviews with families of children who abandoned treatment, we deduced the timeline of uninterrupted retinoblastoma disease progression. We showed that intraocular retinoblastoma progress to orbital extension had a median period of 13.7 months, from orbital disease to systemic metastasis 2.6 months and with only 2.0 months to death. If left untreated, 100% of children died within 48 months from diagnosis. We hope these new findings encourage clinicians and parents to make informed decisions in the management of children with retinoblastoma.

**Abstract:**

Treatment abandonment is a leading cause of death in children with retinoblastoma worldwide. We studied children who abandoned treatment upfront at diagnosis to delineate the natural history of untreated retinoblastoma. Studied were children who received no treatment, diagnosed between 2007 and 2017 at 29 Chinese centers. Data were retrospectively collected from medical chart reviews and interviews with each patient’s family. During the study period, 44 children received no treatment after diagnosis of retinoblastoma. Clinical or radiologic evidence of orbital extension was available for 25 children, and radiologic evidence of systemic metastasis was available for 12 children. Median times from diagnosis of intraocular tumor to orbital disease was 13.7 months, orbital disease to metastasis was 2.6 months, and metastasis to death was 2.0 months. Children with brain metastasis had shorter survival than those with metastasis to other sites (median 1.0 vs. 3.1 months; *p* = 0.015). Overall, 36% of patients died within 12 months of diagnosis, 77% within 24 months, 95% within 36 months and 100% within 48 months. While multiple factors influence refusal of treatment, insights into the natural history of retinoblastoma derived from real-world evidence can inform clinicians and parents that retinoblastoma is life-threatening and encourage urgent treatment at diagnosis.

## 1. Introduction

Retinoblastoma, the most common pediatric intraocular malignancy [1], is one of the most curable cancers, especially with early diagnosis. Simple enucleation is often curative when retinoblastoma is confined inside the eye [2,3]. However, untreated retinoblastoma invades the orbit, metastasizes to the brain and other organs and is uniformly fatal [4]. To avoid enucleation, a multitude of globe salvage modalities are available including systemic chemotherapy [5], intra-arterial chemotherapy [6,7], intravitreal chemotherapy [8,9] brachytherapy [10], external beam radiation [11,12], and recently emerged tumor endoresection via pars plana vitrectomy [13]. Despite good survival and globe prognosis with these therapies, noncompliance is a major cause of death in low- and middle- income countries, where the majority of patients with retinoblastoma reside [14].

Treatment refusal and abandonment in childhood cancer is a complex multifactorial phenomenon influenced by both socio and disease-specific factors. Although enucleation of eyes with retinoblastoma is a life-saving procedure readily available in most settings, the stigma associated with eye removal too often leads parents to refuse or abandon therapy. Low socioeconomic status, long distance to treatment centers and preference for alternative medicine also influences treatment refusal [15,16]. Some families may temporarily abandon treatment and return only after advanced disease has developed, when it is too late for cure. A large retrospective study by Vasquez, L. et al. shows that children diagnosed with retinoblastoma were at a higher risk of treatment abandonment compared to those diagnosed with all other pediatric solid tumors [17]. Improving treatment compliance is an important step to address retinoblastoma mortality worldwide.

The position statement of the International Society of Pediatric Oncology Abandonment of Treatment Working Group [18] defined treatment abandonment as a hiatus of four or more weeks in the scheduled treatment. This definition was further subclassified into failure to begin treatment and failure to continue a planned course of treatment. By retrospectively studying children with retinoblastoma who failed to begin treatment at diagnosis, our aim was to delineate the natural history of untreated retinoblastoma. We hope that this real-world evidence can assist clinicians to show parents the dismal survival prospect of untreated retinoblastoma and thereby facilitate acceptance of treatment.

## 2. Materials and Methods

### 2.1. Eligibility

This was a multicenter retrospective cohort study of all children who received no treatment after diagnosis of retinoblastoma at 29 Chinese treatment centers between 1 April 2007 and 31 July 2017. Children who received any retinoblastoma treatment, including any form of focal therapy, chemotherapy, radiation or surgery following diagnosis were excluded from this study.

### 2.2. Data Collection and Ethics

Clinical information was collected via medical chart reviews and telephone interviews with families of patients to identify events after the last clinical visit (Raw data, Table S1). The first phone call was made to the patient’s family after two scheduled follow-ups were missed. If the child was alive, we called once every year to inquire about the status of the child. The last phone call for this cohort was on 12 August 2019. Preset questions were used in every phone interview. All clinical staging and interviews were done by the same ophthalmologist (J.Z.). For children who were not followed in our clinics, diagnosis of orbital disease and systemic metastasis was based on assessments performed at the child’s local hospital. Orbital disease was defined as clinical, CT or MRI finding of orbital, optic nerve, or full-thickness trans-scleral invasion. Systemic metastasis was defined as MRI or PET detection of tumor invasion of a distant organ outside of the ocular or periocular tissues. Date of orbital extension was defined as the date of orbital tumor diagnosis in a patient without systemic metastasis. Data collected included sex, age at diagnosis, first sign, family history, International Intraocular Retinoblastoma Classification (IIRC [19]) of the eye at diagnosis, location of metastasis and dates of last clinic follow-up, orbital disease, distal metastasis and death. A retrospective review of data without consent was approved by the Ethics Board of Beijing Children’s Hospital.

### 2.3. Statistical Analysis

Data were summarized using frequency/percentage for categorical variables and median/range for continuous variables. Categorical and continuous variables were compared between groups via the Chi-squared test and Mann-Whitney U test, respectively. The Kaplan-Meier method was used to estimate survival. All *p*-values reported are two sided; *p* < 0.05 indicated significance. All analysis was performed using SPSS Version 25 (IBM Corp., New York, NY, USA).

## 3. Results

### 3.1. Study Findings

#### 3.1.1. Patient Characteristics

**Figure 1:**
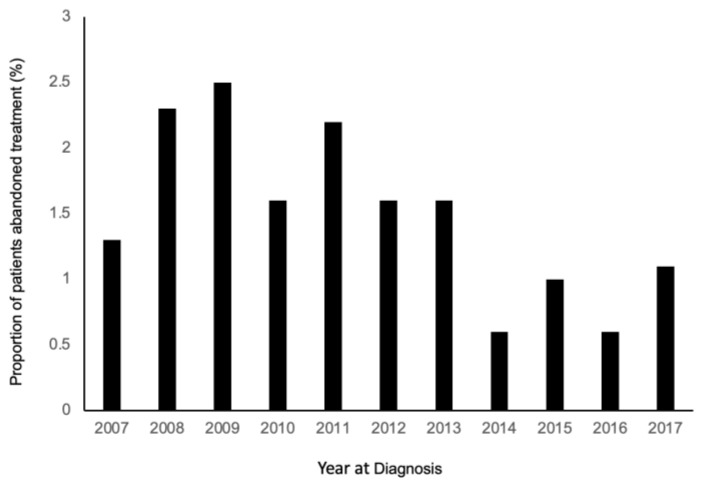
Proportion of patients who abandoned treatment by year of diagnosis from 2007 to 2017.

Of 3780 consecutive children with retinoblastoma managed across 29 treatment centers over a 10-year period, a total of 264 (7%) died, including 44 (1%) who were confirmed to have received no treatment due to treatment abandonment at diagnosis. The proportion of children who abandoned treatment trended down from 2007 to 2017 (Figure 1). Of 44 children who abandoned treatment at diagnosis, 38 (86%) had no subsequent follow-up in clinic, and six (14%) were followed for a median of 6.3 months (range, 2.5–23.2 months).

There were 17 (39%) females and 27 (61%) males (Table 1). Twelve (27%) children had unilateral disease and 32 (73%) had bilateral disease. A total of 76 eyes of 44 children were affected, with six (8%) eyes (six children) presenting at initial diagnosis with extraocular orbital disease. Of 70 eyes presenting with intraocular disease, the IIRC stages were Group A (one, 1%), Group B (six, 9%), Group C (three, 4%), Group D (24, 34%), Group E (32, 46%) and unknown (four, 6%). Two children with bilateral disease (four eyes) abandoned treatment upon confirmed diagnosis of retinoblastoma and refused to be placed under anesthesia for further clinical staging. All 42 children with known clinical staging had at least one eye with Group D, E or orbital disease.

The median age of children at the first sign was 5.0 months (range, 0.2–40.6 months) and median age at diagnosis was 9.0 months (range, 1.0–46.9 months). The median lag time between the first sign and diagnosis was 1.3 months (range, 0–11.6 months). Lag time was not significantly different between children with unilateral vs. bilateral disease (median 2.3 months vs. 1.0 months; *p* = 0.067). The first presenting signs were leukocoria (25, 56%), red eye (seven, 16%), strabismus (three, 7%), epiphora (three, 7%), low vision (two, 5%), buphthalmos (one, 2%), hypopyon (one, 2%) and incidental finding on eye screening (two, 5%). Two (5%) children had family history of retinoblastoma.

#### 3.1.2. Orbital Extension

**Figure 2:**
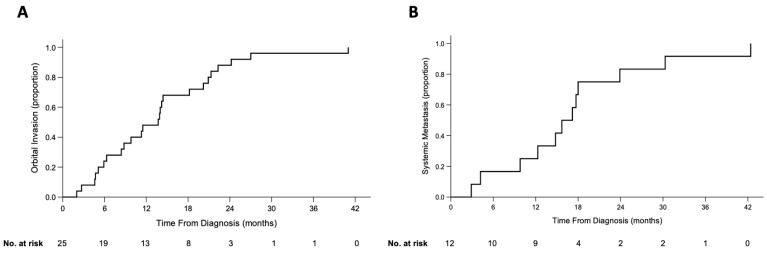
Kaplan-Meier curves of patients who received no treatment: (**A**) 25 patients with known dates of orbital extension, (**B**) 12 patients with known dates of systemic metastasis.

Date of orbital extension from intraocular disease was known for 25/38 (66%) children. The IIRC staging of eyes with clinical/imaging confirmation of orbital extension was Group B (one, 4%), Group D (eight, 32%), Group E (14, 56%), and unknown (two, 8%). Time from diagnosis of intraocular tumor to orbital disease was median 13.7 (range, 2.0–41.0) months (Figure 2A). The median time from diagnosis to orbital extension was not significantly different for Group D (14.8 months) and Group E (11.3 months) eyes (*p* = 0.357).

#### 3.1.3. Metastasis

MRI/PET evidence of systemic metastasis was available for 12/44 children, involving brain (eight), foot (one), skull (one), parotid gland (one), and one child had metastasis to the hand, posterior neck and skull. Time from diagnosis to metastasis was median 14.0 (range, 2.9–23.9) months for brain metastasis and 23.8 (range 14.8–23.9) months for nonbrain metastasis. Time from orbital tumor to distant metastasis was median 2.6 (range, 0.9–4.2) months (Figure 2B).

#### 3.1.4. Death

**Figure 3:**
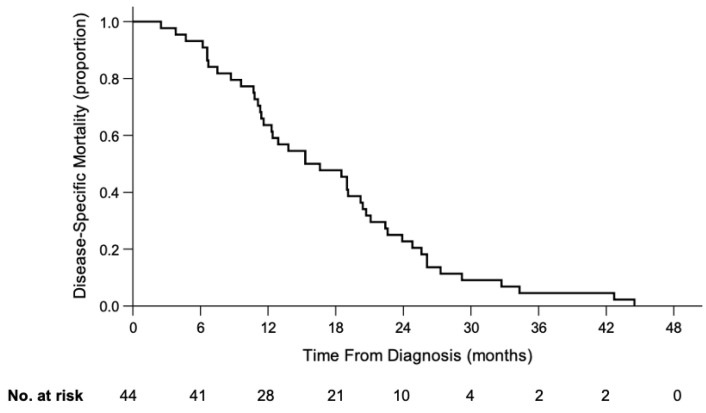
Kaplan-Meier curve of disease-specific survival of 44 patients who received no treatment.

The date of death was known for 44/44 children. Time from diagnosis to death was median 16.0 months (range, 2.5–44.5) (Figure 3); 36% of children died within 12 months from diagnosis, 77% within 24 months, 95% within 36 months and 100% within 48 months. Children with orbital disease at diagnosis had shorter survival times from diagnosis compared to children presenting with intraocular disease (median 8.1 vs. 19.0 months; *p* = 0.004). However, survival time from diagnosis was not significantly different between children with metastasis from IIRC Group D and Group E eyes (median 19.0 vs. 18.4 months; *p* = 0.472). Children who presented with unilateral disease had shorter survival times from diagnosis than those with bilateral disease (median 11.3 vs. 19.1 months; *p* = 0.012). However, a greater proportion of unilateral children had orbital disease at diagnosis compared to bilateral children (33% vs. 6%; *p* = 0.020).

The median time from orbital disease to death was 4.7 (range, 1.7–19.2) months and the median time from metastatic disease to death was 2.0 (range, 0.4–10.1) months. Children with initial brain metastasis had shorter survival times than those with initial nonbrain metastasis (median 1.0 vs. 3.1 months; *p* = 0.015). At the time of death, periorbital tumors were noted in 33/44 children through either clinical examination or imaging; 30 who had prominent proptosis were also noticed by parents or healthcare providers. Overall, 14 (32%) children did not have prominent proptosis at the time of death.

## 4. Discussion

Treatment abandonment condemns patients to adverse outcomes and is a major contributor to the survival disparity between children with cancer in the developing and the developed world [20]. In high-income countries, legal frameworks are often in place to prevent withholding of medically necessary treatments from children. However, in most middle and low-income countries, parents have the legal authority to refuse medical treatment, even those that are necessary to save the child’s life. The present study delineates the natural history of untreated retinoblastoma in 44 Chinese children with retinoblastoma who abandoned treatment at diagnosis. We hope insights from this study may aid physicians to better counsel parents about the mortality consequence of untreated retinoblastoma, facilitating improved compliance.

Of 3780 children diagnosed with retinoblastoma by our treatment over a 10-year period, 1.4% were confirmed to have not received any treatment since diagnosis. The median age at diagnosis for children who abandoned treatment at diagnosis was nine months, significantly younger than previous studies on general clinical presentation of retinoblastoma in China (25 months) [21], Taiwan (26 months) [22], India (29 months) [23], Korea (21 months) [24], and Malaysia (22 months) [25]. The median lag period from first sign to diagnosis was 1.3 months, similar to two Chinese studies which reported lag periods of one month and two months, respectively [21,26]. This suggests that diagnosis of retinoblastoma is timely in China, but that advantage to cure that is lost when treatment at diagnosis is abandoned. The proportion of children with bilateral disease in our cohort was 73%, higher than the 20% and 40% bilateral disease in two other Chinese studies [21,26]. We hypothesize that bilateral disease with poor visual prognosis in both eyes may predispose to treatment abandonment.

Orbital retinoblastoma, commonly associated with delay to seek care, is a devastating diagnosis that generally carries poor prognosis, with mortality ranging from 25–100% [27]. In our case series, 14% had primary orbital retinoblastoma. Gao et al. reported that of children diagnosed with retinoblastoma in southwestern China, 9% were diagnosed with primary orbital disease [21]. In our cohort, all patients had at least one eye with Group D, E or orbital disease, which suggests treatment abandonment mainly affects patients with advanced disease.

While natural disease progression from intraocular tumor to orbital disease to systemic metastasis to death is well-established [4], there is little in current literature about the timing of uninterrupted disease progression. In our cohort, extraocular extension was always unilateral, even in children with bilateral disease. The median time from intraocular disease to clinically observed orbital disease was 13.7 months. Although there is no direct comparison, in a retrospective review of 1674 patients, Kim et al. identified that 4.2% of eyes develop orbital recurrence after enucleation with a mean time from enucleation to orbital recurrence six months [28].

The median time from orbital tumor to metastatic disease was 2.6 months, and another 2.0 months from metastatic disease to death. In our cohort, 30 children had prominent proptosis at the time of death. This is consistent with the observation that direct invasion of the retrobulbar optic nerve was the most common route of metastasis [29]. In the 14 children without proptosis, alternative routes of spread might be subarachnoid fluid or hematogenous dissemination, or death could have been due to pinealoblastoma.

The central nervous system (CNS) has been reported as the most common site of retinoblastoma metastasis, with worse prognosis than metastasis to other distant sites. In other studies of retinoblastoma CNS metastasis, the survival rate was close to 0% despite high dose chemotherapy, radiotherapy and autologous stem cell rescue [30,31,32]. In contrast, metastasis without CNS involvement has a five-year survival of approximately 50% with treatment [30]. Consistent with CNS metastasis carrying a worse prognosis, we observed that median survival time was significantly shorter for CNS metastasis than non-CNS metastasis (median 1.0 vs. 3.1 months; *p* = 0.015). Despite the initial site of metastasis being non-CNS, subsequent brain metastasis still occurred in some patients.

In the present study, we observed that progression from diagnosis to death took a median time of 16.0 months: 19.0 months Group D eyes, 18.4 months Group E eyes and 8.1 months for those who presented with extraocular disease. In our review of 220/3780 (6%) children who were diagnosed in the same time frame (2007–2017), received treatment and died, the median time from diagnosis to death was similar, i.e., 21.4 months for Group D eyes, 16.5 months for Group E eyes and 8.3 months for children with extraocular disease [unpublished]. While treatment prevents deaths for 94% children with retinoblastoma [unpublished], it may not significantly extend the time from diagnosis to death for those with unrecognized or recognized extraocular disease.

As evident in our study, death is the uniform outcome of untreated retinoblastoma. According to our experience and published studies, financial constraint is a common reason for treatment abandonment in developing countries [15]. Since retinoblastoma is a rare disease, specialized care is only available in selected academic institutions, all located in major urban centers. Since 2006, our team based in Beijing routinely travelled to 29 centers across China bringing retinoblastoma care closer to the patients. Furthermore, for families with financial constraints, we offered fundus examination under topical anesthesia which eliminated the costs of blood work, general anesthesia, and hospital admission.

Prior studies have shown that fewer than 20% of enucleated eyes have high-risk histopathologic features, with 50% surviving at a five-year follow-up without adjuvant chemotherapy [3,33]. Therefore, enucleation as a monotherapy is curative in 90% of intraocular retinoblastoma. However, unwillingness to enucleate is a major reason for treatment abandonment [16]. In the AHOPCA II study, children with buphthalmos and considered at risk of therapy abandonment were preselected to receive pre-enucleation chemotherapy [34]. Through this approach, they showed a reduction in treatment abandonment to 4%, an improvement from 16% observed in the AHOPCA I study [35]. A Ugandan study of 270 children showed that those treated with neoadjuvant and adjuvant chemotherapy had a 37% lower risk of dying than those diagnosed in the prechemotherapy era [36]. Waddle et al. noted that pre-enucleation chemotherapy enabled parents to interact with families of enucleated children and decrease the likelihood of refusal of subsequent enucleation. For children with a high risk of treatment abandonment, chemotherapy may be helpful to families who temporarily refuse enucleation while active counselling is offered. However, delay to enucleation because of pre-enucleation chemotherapy is associated with worse survival than upfront primary enucleation, and is best avoided in eyes with no visual potential or low likelihood of successful salvage [3].

A limitation of our study is the reliance on the parents’ account, which may be subjected to recall bias. In addition, due to lack of follow-up at regular intervals, we were likely unable to capture the earliest onset of orbital extension and systemic metastasis. This may have contributed to underestimation of the aggressiveness of disease progression from diagnosis to orbital extension to metastasis. Nevertheless, time from diagnosis to death was unambiguous.

## 5. Conclusions

Disease natural history is assessed by following a cohort of patients who receive no therapeutic intervention. Nonintervention or observation, while applicable in some cancers (e.g., low-risk prostate cancer), is not a constructive option in retinoblastoma, an aggressive but highly curable cancer. Furthermore, patients who forgo treatment against medical advice are also difficult to study because of cessation of regular follow-up within the healthcare system. To bypass these challenges, we employed a novel approach of systematically calling families of children who abandoned treatment to acquire information on this previously unstudied cohort. This approach enabled us to generate new insights on uninterrupted progression of retinoblastoma, an unacceptable but common reality in developing countries like China. We hope this knowledge will support clinicians and parents to make informed decisions about the management of children with retinoblastoma, especially in low-resource countries.

## Figures and Tables

**Table 1:** Clinical characteristics of 44 patients who refused any treatment.

Characteristic		Number of Patients	%
Sex	Male	17	39
	Female	27	61
Laterality	Unilateral	12	27
	Bilateral	32	73
Stage of RB at Presentation	Intraocular	6	14
	Extraocular	38	86
Family History of RB	No	42	95
	Yes	2	5
Year of Diagnosis	2007–2009	13	29
	2010–2012	15	34
	2013–2015	10	23
	2016–2017	6	14
Age at first sign (months)	Median (range)	5.0 (0.2–40.6)	
Age at diagnosis (months)	Median (range)	9.0 (1.0–46.9)	
Lag period (months)	Median (range)	1.3 (0–11.6)	

RB, retinoblastoma.

## Data Availability

Raw data are available as a supplementary file.

## Supplementary Materials

The following are available online at https://www.mdpi.com/article/10.3390/cancers13153646/s1, Table S1: Raw Data.
